# Multivariate meta-analysis of critical care meta-analyses: a meta-epidemiological study

**DOI:** 10.1186/s12874-021-01336-4

**Published:** 2021-07-18

**Authors:** John L. Moran

**Affiliations:** Department of Intensive Care Medicine, The Queen Elizabeth Hospital, Woodville, SA 5011 Australia

**Keywords:** Multivariate meta-analysis, Critical care, Random effects, Borrowing of strength, Metric

## Abstract

**Background:**

Meta-analyses typically consider multiple outcomes and report univariate effect sizes considered as independent. Multivariate meta-analysis (MVMA) incorporates outcome correlation and synthesises direct evidence and related outcome estimates within a single analysis. In a series of meta-analyses from the critically ill literature, the current study contrasts multiple univariate effect estimates and their precision with those derived from MVMA.

**Methods:**

A previous meta-epidemiological study was used to identify meta-analyses with either one or two secondary outcomes providing sufficient detail to structure bivariate or tri-variate MVMA, with mortality as primary outcome. Analysis was performed using a random effects model for both odds ratio (OR) and risk ratio (RR); borrowing of strength (BoS) between multivariate outcome estimates was reported. Estimate comparisons, β coefficients, standard errors (SE) and confidence interval (CI) width, univariate versus multivariate, were performed using Lin’s concordance correlation coefficient (CCC).

**Results:**

In bivariate meta-analyses, for OR (*n* = 49) and RR (*n* = 48), there was substantial concordance (≥ 0.69) between estimates; but this was less so for tri-variate meta-analyses for both OR (*n* = 25; ≥ 0.38) and RR (≥ -0.10; *n* = 22). A variable change in the multivariate precision of primary mortality outcome estimates compared with univariate was present for both bivariate and tri-variate meta-analyses and for metrics. For second outcomes, precision tended to decrease and CI width increase for bivariate meta-analyses, but was variable in the tri-variate. For third outcomes, precision increased and CI width decreased. In bivariate meta-analyses, OR coefficient significance reversal, univariate versus MVMA, occurred once for mortality and 6 cases for second outcomes. RR coefficient significance reversal occurred in 4 cases; 2 were discordant with OR. For tri-variate OR meta-analyses reversal of coefficient estimate significance occurred in two cases for mortality, nine cases for second and 7 cases for third outcomes. In RR meta-analyses significance reversals occurred for mortality in 2 cases, 6 cases for second and 3 cases for third; there were 7 discordances with OR. BoS was greater in trivariate MVMAs compared with bivariate and for OR versus RR.

**Conclusions:**

MVMA would appear to be the preferred solution to multiple univariate analyses; parameter significance changes may occur. Analytic metric appears to be a determinant.

## Background

Meta-analyses typically consider more than one outcome, and the conventional approach is to report multiple univariate effect size estimates of these separate outcomes. Such an approach has two attendant consequences; it ignores the effect of outcome correlation upon individual estimates, assuming that they are independent [1], and engenders multiplicity of the Type I error rate [2]. Confounding such effects is the selective reporting of outcomes, or outcome reporting bias (ORB), whereby secondary outcomes are selectively reported based upon outcome results [3, 4]. Multivariate meta-analysis (MVMA), whereby direct evidence and results from related outcomes are synthesised to yield a summary outcome result [5–7], is an elegant solution to the above problems.

In meta-analyses of interventions in the critically ill, where mortality is a common primary outcome, it would be expected that secondary outcomes such as intensive care unit (ICU) and hospital length of stay, infections and the requirement for mechanical ventilation would demonstrate substantial correlation [6], and with the primary mortality event. MVMA in such meta-analyses would allow joint inference upon multiple outcomes and be of relevance from a methodological and clinical viewpoint. Price et al. suggested that where multiple outcomes routinely occur, MVMA would be “…more likely to have an impact” [8]. From a previous study which reported mortality outcome of a series of meta-analyses in the critically ill [9] utilising only randomised controlled trials, a meta-analytic cohort was identified where secondary outcomes were reported in such detail as to yield bivariate or tri-variate data structures. Tri-variate data structures have been rarely subjected to MVMA; in the Price et al. analysis [8], only one such MVMA was reported. Univariate and multivariate analyses were undertaken and compared with respect to differences between estimated outcome variable coefficients, their standard errors (SE) and 95% confidence interval (CI) width and statistical significance, with no selection of meta-analyses based upon the number of RCTs per meta-analysis. As a by-product of MVMA coefficient estimation, variable correlations, direct information and borrowing of strength (BoS) were determined. Whereas direct information describes the contribution of data from the same outcome, BoS represents the contribution of data from all other outcomes [10, 11]. One problematic requirement of MVMA is the provision of with-study correlations which are rarely reported, although methods based upon individual patient [12] or aggregated data [13] and within the Bayesian framework [14] have been undertaken. Any recommendation for the practical application of MVMA must be accompanied by appropriate software. As such, the “alternative” MVMA model of Riley [15] was employed, whereby an overall correlation, the total marginal correlation between outcomes, was modelled, enabling seamless application to all meta-analyses considered. As results based upon indices of risk, odds ratio (OR) and risk ratio (RR), are not generally inter-translatable [16], both OR and RR estimates were compared.

## Methods

### Ethics

The data for this study was abstracted from published studies and an Ethics clearance was not appropriate.

### Data management

A previous study [9] was used to identify meta-analyses with either one or two secondary outcomes that provided sufficient detail to generate a bivariate or tri-variate MVMA data structure, with mortality as the primary outcome; all meta-analyses were of randomised controlled trials (RCT). Usable second and third outcomes were identified as presented in the original meta-analysis.

### Statistical analysis

1. To facilitate rapid data processing over a large number of models, initial univariate meta-analytic point estimates and standard errors (SE) were computed within Stata™ V17 [17] using the “meta” suite of commands [18]; default estimation used restricted maximum likelihood (REML [19]).
2. Subsequently, both univariate and multivariate outcomes were estimated using the user written Stata command “mvmeta” ([20], Version 3.2.0 6apr2018) in a random effects (RE) formulation. Estimation employed REML with an unstructured covariance and the Broyden-Fletcher-Goldfarb-Shanno (BFGS) algorithm for likelihood maximisation or the Davidon-Fletcher-Powell (DFP) algorithm if there were convergence difficulties. Maximisation employed the “difficult” option (use a different stepping algorithm in nonconcave regions) provided by Stata™.
  (i) The two sets of univariate estimates were subsequently compared.
  (ii) Under persistent convergence difficulties of “mvmeta”, the model was refit assuming the overall correlation matrix was fixed and known, with values set equal to the estimates from either the BGFS or DFP algorithm using the “bscovariance” option of “mvmeta” ([8], Appendix 4).
  (iii) In the MVMA note was taken of very small β coefficient standard errors (SE) with consequent large *z* values for coefficient significance and very small p-values and CI width, such that the estimates were implausible.
  (iv) To avoid the requirement for specific within study correlations [1], the “alternative” model of Riley was used [15], whereby an overall correlation, the total marginal correlation between outcomes [21], was modelled; that is an amalgam of the within and between-study correlations [6, 8]. The reported correlation(s) in this paper were these overall correlation(s) [22].
  (v) Direct information and BoS between estimates were also reported [8, 10, 11], using the default (“sd”) method of “mvmeta”. BoS may be conceptualised as a comparison of variances of the estimated *r*th component of β under the uni- and multivariate models $$BoS_{r}^{{RV}} = 1 - \frac{{{\text{var}}\left( {\hat{\beta }_{{mv,r}} } \right)}}{{{\text{var}}\left( {\hat{\beta }_{{uv,r}} } \right)}}$$, where *RV* refers to relative variance [11]. This ratio has also been described as the efficiency, “E” [10, 23]. An equivalent but alternative method, decomposition of the score function for β, has advantage in that it defines appropriate study weights within an MVMA [11]. In a univariate RE meta-analysis study weights are inversely proportional to the sum of the within- and between- study variances. In an MVMA analysis, as undertaken by “mvmeta”, weights were derived using the score decompensation method, where the score function $$S\left( \theta \right)$$ is the first derivative of the log-likelihood function $$l\left( \theta \right);$$$$S\left( \theta \right) = \frac{{dl\left( \theta \right)}}{{d\theta }}$$ and $$l(\theta )$$ is the likelihood. The weights were broken down into direct information, the contribution of data from the same outcome, and BoS, the contribution of data for all other outcomes. For a univariate analysis, the weights sum to 1, or when expressed as a percentage, 100, as in the “mvmeta” output. In a MVMA, a simple tabulation of direct information and BoS will sum to 100 for each outcome. In particular, the methodology takes the variance components as fixed and the precisions of the point estimates from a MVMA have an expectation of being greater than or equal to those from separate univariate meta-analyses [24], albeit the latter study employed the methods of Van Houwelingen et al. [25] using “Proc Mixed’’ with SAS statistical software, not the “Riley” method [15].
    - The use of the “Riley” method [15] excluded the computation of multivariate *I*^*2*^ for each outcome.
3. The reported confounding effect of small study effects upon changes of statistical significance between univariate and MVMA [5, 8] was explored by inspection of contour-enhanced funnel plots [26] and formal regression based tests, in particular, the Harbord (for binary outcomes) and Egger (for continuous outcomes) tests [27]. Small study effects were reported for all meta-analyses, as a matter of complete reporting, but suffer from the problem of multiple testing. The power and interpretation of the tests are problematic for small RCT number (< 10) meta-analyses and in the presence of moderate (see 4., below) heterogeneity [28, 29]. More importantly, univariate tests may be underpowered compared with the recently described multivariate small study effect test (MSSET), a multivariate extension of Egger’s regression test [30].
4. Meta-analytic heterogeneity was reported as the I^2^ index and adjudged as medium and high if I^2^ ≥ 50 and 75% respectively. The I^2^ index was preferred, compared with τ^2^, as it is comparable across different metrics and number of RCTs [31].
5. The analyses, using frequentist methods, were performed for bivariate and tri-variate models with both OR and RR metrics.
6. Agreement or otherwise between univariate and multivariate estimation results was undertaken using Lin’s concordance correlation coefficient (CCC) via the user written Stata command “concord” [32]. The CCC combines measures of both precision and accuracy to determine how far the observed data deviate from the line of perfect concordance (that is, the line at 45 degrees on a square scatterplot). Other measure to characterise the comparison were:
  (i) Estimate differences average and standard deviation (SD); univariate versus MVMA.
  (ii) 95% (Bland and Altman) limits of agreement (LOA)
  (iii) An F test (Bradley-Blackwood) of equality of means and variances; non-significance implies concordance.
7. Boxplots [33] were used to visualise the density distribution of BOS and total marginal correlations of both OR and RR for bivariate and tri-variate models.

Statistical significance was ascribed at *p* < 0.05.

## Results

The cohort was composed of forty-nine meta-analyses, 18% nutritional therapeutic, 18% non-pharmaceutical therapeutic and 64% pharmaceutical therapeutic, published between 2002 and 2018. The primary outcome in all was mortality; forty-nine were bivariate in outcome data composition and 30 were tri-variate. Details of the mortality, second and third outcome meta-analyses are shown in Tables 1, 2 and 3, respectively. Heterogeneity, as the I^2^ index, of ≥ 50 and ≥ 75% was found in mortality, second and third outcomes in 12, 31 and 50%, and 0, 16 and 23%, respectively. Of the 49 mortality meta-analyses, five [38, 43, 53, 60, 66] demonstrated evidence of small study effects on formal testing (*p* < 0.05); for the second outcome, five [37, 53, 59–61]; and for the third, five [37, 49, 67, 68, 78]. The disparity between the formal test of small study effects (*p* < 0.05) and the increased frequency of “query” for contour-enhanced funnel plot assessment in second and third outcomes versus mortality outcome (37, 40 and 4%, respectively) was noted and may be a function of the power of the test (see Methods, 3.). There was uniform agreement (to the second or third decimal point) between univariate estimates of “mvmeta” and “meta” in Stata™.

**Table 1:** Details of primary (mortality) outcome for meta-analyses

Meta_analysis	Reference	Study	Second_outcome	Third_outcome	Year	No. RCTs mortality	Total patients	Total events	I^2	Mortality sse graphics	Mortality sse test
Griesdale	[34]	Bivariate	Hypoglcaemia		2009	26	13,572	3362	52.25	ok	0.610
Annane	[35]	Bivariate	Shock reversal		2009	20	2384	840	52.48	ok	0.090
Bangalore	[36]	Trivariate	Non-fatal MI	Non-fatal stroke	2008	23	11,862	304	9.23	ok	0.720
Marik	[37]	Trivariate	Infection	Hospital LOS	2008	13	2553	689	57.30	ok	0.990
Marik	[37]	Trivariate	Hospital LOS	Infection	2008	4	144	27	63.99	ok	0.090
Marik	[37]	Trivariate	Hospital LOS	Infection	2008	7	300	26	0.00	ok	0.750
Chan	[38]	Trivariate	VAP	MV length	2007	11	3242	553	37.03	ok	0.010
Gonzalez	[39]	Bivariate	Rebleeding		2008	18	1304	198	0.00	ok	0.470
Ho	[40]	Trivariate	Pneumonia	ICULOS	2006	8	517	125	0.00	ok	0.430
Ho	[41]	Bivariate	ICU LOS		2008	14	1184	299	0.00	ok	0.460
Siempos	[42]	Bivariate	VAP		2010	11	2014	514	0.00	ok	0.990
Singh	[43]	Bivariate	Pneumonia		2009	11	7514	582	1.05	ok	0.040
Peterson	[44]	Bivariate	Neurological outcome		2008	8	781	208	28.16	ok	0.250
Silvestri	[45]	Bivariate	infection		2007	31	4747	1003	3.23	ok	0.990
Whitlock	[46]	Trivariate	New Atrial fibrillation	Bleed post-operative	2008	16	2038	65	0.00	ok	0.190
Piccini	[47]	Trivariate	CVS death	Cardiomyopathy	2009	15	8522	715	0.00	ok	0.150
Landoni	[48]	Bivariate	MI		2010	27	3350	659	11.93	ok	0.160
Brar	[49]	Trivariate	MI	TVR	2009	13	7352	288	0.00	ok	0.170
Landoni	[50]	Trivariate	Acute kidney injury	Hospital LOS	2007	11	1118	191	0.00	ok	0.635
Mazaki	[51]	Bivariate	Infection		2008	15	1832	65	0.00	ok	0.250
Masip	[52]	Bivariate	Intubation		2005	9	468	78	0.00	ok	0.870
Oldani	[53]	Trivariate	Infection	Hospital LOS	2015	24	2834	797	22.22	query	0.020
Szakmany	[54]	Trivariate	VAP	MV length	2015	14	2406	747	0.00	ok	0.360
Alkhawaja	[55]	Trivariate	Pneumonia	ICU LOS	2015	11	977	219	0.00	ok	0.970
Van Zanten	[56]	Trivariate	Infection	Hospital LOS	2015	10	1022	163	0.00	ok	0.240
Wan	[57]	Bivariate	Hospital LOS	MV length	2015	9	706	188	0.00	ok	0.380
Teo	[58]	Bivariate	Clinical response		2014	13	905	96	0.00	ok	0.320
Manzanares	[59]	Trivariate	Infection	MV length	2012	21	2485	631	22.75	ok	0.960
Tian	[60]	Trivariate	Pneumonia	Hospital LOS	2018	16	3225	1110	57.20	query	0.043
Rhodes	[61]	Bivariate	Cinical cure		2018	17	3220	534	0.00	ok	0.060
Nunez-Patino	[62]	Trivariate	Neurological outcome	ICU LOS	2018	10	1361	308	0.00	ok	0.680
Kawano-Dourado	[63]	Bivariate	RRT		2018	10	3665	336	0.00	ok	0.960
Dallimore	[64]	Bivariate	ICU LOS		2018	13	1781	473	27.74	ok	0.340
Chong	[65]	Bivariate	Myocardial infarction		2018	27	10,740	1148	12.66	ok	0.440
Yang	[66]	Trivariate	Renal function recovery	RRT time	2017	9	1636	682	62.70	ok	0.020
Osadnik	[67]	Trivariate	Hospital LOS	MV	2017	12	854	119	0.00	ok	0.667
Lu	[68]	Trivariate	ICU LOS	MV length	2017	16	1179	297	7.35	ok	0.260
Chen	[69]	Trivariate	ICU LOS	RRT	2017	17	2754	198	6.16	ok	0.150
Qureshi	[70]	Bivariate	Acute kidney injury		2016	29	14,167	3321	31.86	ok	0.950
Elke	[71]	Trivariate	Infection	Hospital LOS	2016	16	3167	1004	0.00	ok	0.540
Parikh	[72]	Trivariate	Hospital LOS	ICU LOS	2016	16	3473	913	5.31	ok	0.990
Davies	[73]	Trivariate	VAP	Hospital LOS	2017	14	3238	711	19.75	ok	0.880
Abroug	[74]	Bivariate	Clinical response		2014	9	1097	79	0.00	ok	0.340
Manzanares	[75]	Bivariate	infection		2016	17	1638	340	0.00	ok	0.740
Peter	[76]	Trivariate	Mechanical ventilation	Hospital LOS	2002	15	793	138	0.00	ok	0.195
Wang	[77]	Trivariate	Rebleeding	Surgery	2010	6	1052	21	0.00	ok	0.950
Tao	[78]	Trivariate	Infection	MV length	2016	10	780	208	0.00	ok	0.840
Tang	[79]	Trivariate	Antibiotic exposure	ICU LOS	2009	7	1458	131	0.00	ok	0.410
Muscedere	[80]	Trivariate	VAP	MV length	2011	6	1682	341	0.00	ok	0.580

*LOS* Length of stay (days), *ICU* Intensive care unit, *VAP* Ventilator associated pneumonia, *CVS* Cardiovascular, *MV* Mechanical ventilation, *TVR* Target vessel revascularization, *RRT* Renal replacement therapy, *MI* Myocardial infarction, *Ok* Visual assessment of contour-enhanced revealed no problematic asymmetry, *query* Visual assessment of contour-enhanced revealed problematic asymmetry, *sse* Small study effects

**Table 2:** Details of second outcomes for meta-analyses

Meta_analysis	Reference	Study	Second_outcome	Third_outcome	Year	Second outcome RCT no.	Second outcome total patients	Second outcome total events	I^2	Second outcome sse graphics	Second outcome sse test
Griesdale	[34]	Bivariate	Hypoglcaemia		2009	14	12,337	752	38.15	query	0.340
Annane	[35]	Bivariate	Shock reversal		2009	7	1368	708	67.95	query	0.230
Bangalore	[36]	Trivariate	Non-fatal MI	Non-fatal stroke	2008	20	11,734	436	0.00	query	0.052
Marik	[37]	Trivariate	Infection	Hospital LOS	2008	9	1828	725	35.34	query	0.008
Marik	[37]	Trivariate	Hospital LOS	Infection	2008	4	147	N/A	60.69	ok	0.370
Marik	[37]	Trivariate	Hospital LOS	Infection	2008	5	227	N/A	87.00	ok	0.150
Chan	[38]	Trivariate	VAP	MV length	2007	7	300	120	57.44	query	0.130
Gonzalez	[39]	Bivariate	Rebleeding		2008	18	1304	409	46.29	ok	0.670
Ho	[40]	Trivariate	Pneumonia	ICULOS	2006	4	216	42	1.88	ok	0.800
Ho	[41]	Bivariate	ICU LOS		2008	7	454	N/A	76.86	ok	0.550
Siempos	[42]	Bivariate	VAP		2010	10	1958	310	0.00	ok	0.470
Singh	[43]	Bivariate	Pneumonia		2009	11	7514	465	36.50	ok	0.250
Peterson	[44]	Bivariate	Neurological outcome		2008	8	781	357	57.37	query	0.540
Silvestri	[45]	Bivariate	infection		2007	31	4747	625	26.61	ok	0.550
Whitlock	[46]	Trivariate	New Atrial fibrillation	Bleed post-operative	2008	8	1090	332	0.00	ok	0.240
Piccini	[47]	Trivariate	CVS death	Cardiomyopathy	2009	14	8244	1252	1.27	ok	0.050
Landoni	[48]	Bivariate	MI		2010	14	849	17	0.00	ok	0.576
Brar	[49]	Trivariate	MI	TVR	2009	13	7352	261	0.00	ok	0.471
Landoni	[50]	Trivariate	Acute kidney injury	Hospital LOS	2007	9	1037	242	0.00	query	0.917
Mazaki	[51]	Bivariate	Infection		2008	8	1250	252	16.87	ok	0.497
Masip	[52]	Bivariate	Intubation		2005	9	468	192	0.00	ok	0.728
Oldani	[53]	Trivariate	Infection	Hospital LOS	2015	12	1941	1158	75.88	query	0.006
Szakmany	[54]	Trivariate	VAP	MV length	2015	6	1048	389	54.60	query	0.114
Alkhawaja	[55]	Trivariate	Pneumonia	ICU LOS	2015	8	801	189	8.41	ok	0.778
Van Zanten	[56]	Trivariate	Infection	Hospital LOS	2015	4	776	296	0.00	ok	0.085
Wan	[57]	Bivariate	Hospital LOS	MV length	2015	4	217	N/A	99.47	query	0.354
Teo	[58]	Bivariate	Clinical response		2014	4	814	639	0.00	ok	0.278
Manzanares	[59]	Trivariate	Infection	MV length	2012	10	1742	449	0.00	ok	0.663
Tian	[60]	Trivariate	Pneumonia	Hospital LOS	2018	9	2994	354	37.90	query	0.001
Rhodes	[61]	Bivariate	Cinical cure		2018	10	1935	1315	47.80	query	0.016
Nunez-Patino	[62]	Trivariate	Neurological outcome	ICU LOS	2018	5	981	598	59.04	ok	0.080
Kawano-Dourado	[63]	Bivariate	RRT		2018	7	3463	125	0.00	ok	0.483
Dallimore	[64]	Bivariate	ICU LOS		2018	6	1069	N/A	0.00	ok	0.249
Chong	[65]	Bivariate	Myocardial infarction		2018	13	7269	136	17.31	ok	0.427
Yang	[66]	Trivariate	Renal function recovery	RRT time	2017	6	1292	43	0.00	ok	0.625
Osadnik	[67]	Trivariate	Hospital LOS	MV	2017	9	869	N/A	9.70	ok	0.442
Lu	[68]	Trivariate	ICU LOS	MV length	2017	11	865	N/A	86.68	ok	0.439
Chen	[69]	Trivariate	ICU LOS	RRT	2017	10	1746	N/A	91.38	ok	0.726
Qureshi	[70]	Bivariate	Acute kidney injury		2016	7	8203	1012	0.00	query	0.206
Elke	[71]	Trivariate	Infection	Hospital LOS	2016	9	2768	521	47.95	query	0.060
Parikh	[72]	Trivariate	Hospital LOS	ICU LOS	2016	7	830	N/A	86.83	query	0.751
Davies	[73]	Trivariate	VAP	Hospital LOS	2017	6	2064	1720	53.03	query	0.536
Abroug	[74]	Bivariate	Clinical response		2014	9	1097	764	0.00	query	0.383
Manzanares	[75]	Bivariate	infection		2016	8	761	292	42.79	query	0.031
Peter	[76]	Trivariate	Mechanical ventilation	Hospital LOS	2002	12	695	214	16.56	ok	0.948
Wang	[77]	Trivariate	Rebleeding	Surgery	2010	6	1052	86	0.00	ok	0.747
Tao	[78]	Trivariate	Infection	MV length	2016	5	457	118	0.00	ok	0.143
Tang	[79]	Trivariate	Antibiotic exposure	ICU LOS	2009	6	1386	N/A	97.11	ok	0.828
Muscedere	[80]	Trivariate	VAP	MV length	2011	6	1682	212	0.00	ok	0.266

*LOS* Length of stay (days), *ICU* Intensive care unit, *VAP* Ventilator associated pneumonia, *CVS* Cardiovascular, *MV* Mechanical ventilation, *TVR* Target vessel revascularization, *RRT* Renal replacement therapy, *MI* Myocardial infarction, *Ok* Visual assessment of contour-enhanced revealed no problematic asymmetry, *query* Visual assessment of contour-enhanced revealed problematic asymmetry

**Table 3:** Details of third outcomes for meta-analyses

Meta_analysis	Reference	Study	Second_outcome	Third_outcome	Year	Third outcome RCT no.	Third outcome total patients	Third outcome total events	I^2	Third outcome sse graphics	Third outcome sse test
Griesdale	[34]	Bivariate	Hypoglcaemia		2009						
Annane	[35]	Bivariate	Shock reversal		2009						
Bangalore	[36]	Trivariate	Non-fatal MI	Non-fatal stroke	2008	13	11,233	55	0.00	ok	0.260
Marik	[37]	Trivariate	Infection	Hospital LOS	2008	4	835	N/A	76.21	query	0.002
Marik	[37]	Trivariate	Hospital LOS	Infection	2008	4	144	74	0.00	ok	0.596
Marik	[37]	Trivariate	Hospital LOS	Infection	2008	7	300	120	60.37	ok	0.710
Chan	[38]	Trivariate	VAP	MV length	2007	5	1597	N/A	23.90	ok	0.400
Gonzalez	[39]	Bivariate	Rebleeding		2008						
Ho	[40]	Trivariate	Pneumonia	ICULOS	2006	5	336		0.00	ok	0.410
Ho	[41]	Bivariate	ICU LOS		2008						
Siempos	[42]	Bivariate	VAP		2010						
Singh	[43]	Bivariate	Pneumonia		2009						
Peterson	[44]	Bivariate	Neurological outcome		2008						
Silvestri	[45]	Bivariate	infection		2007						
Whitlock	[46]	Trivariate	New Atrial fibrillation	Bleed post-operative	2008	4	513	N/A	7.89	ok	0.120
Piccini	[47]	Trivariate	CVS death	Cardiomyopathy	2009	15	8522	1607	3.39	ok	0.228
Landoni	[48]	Bivariate	MI		2010						
Brar	[49]	Trivariate	MI	TVR	2009	13	7352	561	35.99	query	0.001
Landoni	[50]	Trivariate	Acute kidney injury	Hospital LOS	2007	8	695	N/A	0.00	ok	0.693
Mazaki	[51]	Bivariate	Infection		2008						
Masip	[52]	Bivariate	Intubation		2005						
Oldani	[53]	Trivariate	Infection	Hospital LOS	2015	15	2297	N/A	59.35	query	0.710
Szakmany	[54]	Trivariate	VAP	MV length	2015	9	1623	N/A	76.73	query	0.532
Alkhawaja	[55]	Trivariate	Pneumonia	ICU LOS	2015	6	484	N/A	17.63	ok	0.208
Van Zanten	[56]	Trivariate	Infection	Hospital LOS	2015	6	535	N/A	61.28	query	0.540
Wan	[57]	Bivariate	Hospital LOS	MV length	2015	7	617	N/A	88.84	query	0.562
Teo	[58]	Bivariate	Clinical response		2014						
Manzanares	[59]	Trivariate	Infection	MV length	2012	5	368	N/A	65.48	query	0.510
Tian	[60]	Trivariate	Pneumonia	Hospital LOS	2018	8	517	N/A	12.46	ok	0.624
Rhodes	[61]	Bivariate	Cinical cure		2018						
Nunez-Patino	[62]	Trivariate	Neurological outcome	ICU LOS	2018	5	763		0.00	ok	0.194
Kawano-Dourado	[63]	Bivariate	RRT		2018						
Dallimore	[64]	Bivariate	ICU LOS		2018						
Chong	[65]	Bivariate	Myocardial infarction		2018						
Yang	[66]	Trivariate	Renal function recovery	RRT time	2017	3	576		97.92	query	0.003
Osadnik	[67]	Trivariate	Hospital LOS	MV	2017	12	846	196	84.57	query	0.043
Lu	[68]	Trivariate	ICU LOS	MV length	2017	7	495	N/A	67.15	ok	0.680
Chen	[69]	Trivariate	ICU LOS	RRT	2017	8	1908	129	0.00	ok	0.413
Qureshi	[70]	Bivariate	Acute kidney injury		2016						
Elke	[71]	Trivariate	Infection	Hospital LOS	2016	6	2684	N/A	0.00	ok	0.686
Parikh	[72]	Trivariate	Hospital LOS	ICU LOS	2016	10	2782	421	50.85	ok	0.396
Davies	[73]	Trivariate	VAP	Hospital LOS	2017	10	2747	N/A	72.54	ok	0.340
Abroug	[74]	Bivariate	Clinical response		2014						
Manzanares	[75]	Bivariate	infection		2016						
Peter	[76]	Trivariate	Mechanical ventilation	Hospital LOS	2002	10	398	N/A	88.06	query	0.246
Wang	[77]	Trivariate	Rebleeding	Surgery	2010	5	949	22	0.00	ok	0.570
Tao	[78]	Trivariate	Infection	MV length	2016	4	288	N/A	52.89	query	0.036
Tang	[79]	Trivariate	Antibiotic exposure	ICU LOS	2009	6	1386	N/A	99.01	query	0.674
Muscedere	[80]	Trivariate	VAP	MV length	2011	4	458	N/A	6.27	ok	0.412

*LOS* Length of stay (days), *ICU* Intensive care unit, *VAP* Ventilator associated pneumonia, *CVS* Cardiovascular, *MV* Mechanical ventilation, *TVR* Target vessel revascularization, *RRT* Renal replacement therapy, *MI* Myocardial infarction, *Ok* Visual assessment of contour-enhanced revealed no problematic asymmetry, *query* Visual assessment of contour-enhanced revealed problematic asymmetry

### Bivariate model: OR

For the 49 meta-analyses, median (minimum, p25, p75, maximum) number of RCT per meta-analysis for the primary mortality outcome was 13(4, 10, 17, 31); for the second outcomes, 8(4, 6, 10, 31); see Tables 1 and 2. In only 11 meta-analyses was there equality between the reported primary and secondary outcome study numbers. In the MVMA the “bscovariance” option was used once only and there were no instances of “large” *Z* values. Second outcomes were binary in 39 and continuous in 10 (Tables 1 and 2). Estimate analysis is given in Table 4. Across all outcomes and estimates, the concordance, univariate versus multivariate, was substantial, with a general relative increment, albeit uneven, in the magnitude of multivariate estimates. Means and variances demonstrated little concordance. Reversal of coefficient estimate significance, univariate versus MVMA, occurred no cases for mortality and 6 cases for second outcomes (significant to non-significant in five [36, 43, 70, 71, 79], one meta-analysis exhibiting small study effects [43]; non-significant to significant in in one [59]).

**Table 4:** Concordance analysis for bivariate model (OR): univariate versus multivariate

	**CCC (95%CI)**	**Difference (SD)**	**95%LOA**	**B-B F-test**
**OR**				
**Mortality**				
β	0.915 (0.871, 0.958)	-0.012 (0.065)	-0.319, 0.115	0.016
Mortality SE	0.807 (0.743, 0.871)	-0.017 (0.101)	-0.125, 0.181	0.0001
CI width	0.782 (0.734, 0.831)	-0.133 (0.718)	-1.540, 1.274	0.0001
**Second outcome: Binary (*n* = 39)**				
β	0.987 (0.980,0.995)	-0.005 (0.177)	-0.353, 0.342	0.270
SE	0.919 (0.874, 0.964	-0.033 (0.080)	-0.189, 0.123	0.0001
CI width	0.826 (0.731, 0.920)	-0.240 (0.496)	-1.213, 0.732	0.002
**Second outcome: Continuous (*n* = 10)**				
β	0.993 (0.977, 0.998)	-0.007 (0.626)	-1.235, 1.220	0.413
SE	0.960 (0.917, 0.987)	-0.319 (0.663)	-1.559, 0.921	0.041
CI width	0.960 (0.915, 0.987)	-1.319 (2.457)	-6.134, 3.496	0.044

*CCC* Concordance correlation coefficient, *LOA* Bland and Altman limits of agreement, *B-B* Bradley-Blackwood

### Bivariate model: RR

In the MVMA, 49 meta-analyses were considered and there were no instances of “large” *Z* values. Concordance estimate analysis is given in Table 5. Substantial concordance was seen between uni- and multivariate estimates, with a variable relative increment of multivariate estimates (SE and CI width) across outcomes. Multivariate β estimates were variable with respect to univariate and means and variances lacked concordance. Reversal of coefficient estimate significance, univariate versus MVMA, occurred in one case for mortality outcome (significant to non-significant, [52]) and 3 cases for second outcomes (significant to non-significant [36, 61, 70]; one instance [61] was discordant with the OR metric and one instance exhibiting small study effects [61]).

**Table 5:** Concordance analysis for bivariate model (RR): univariate versus multivariate

	**CCC (95%CI)**	**Difference (SD)**	**95%LOA**	**B-B F-test**
**RR**				
**Mortality**				
β	0.972 (0.956, 987)	-0.005 (0.028)	-0.061, 0.051	0.071
Mortality SE	0.692 (0.550, 0.834)	0.012 (0.063)	-0.013, 0.136	0.074
CI width	0.865 (0.799, 0.932)	-0.010 (0.180)	-0.363, 0.342	0.025
**Second outcome: Binary (*n* = 39)**				
β	0.979 (0.965, 0.992)	0.033 (0.175)	-0.311, 0.376	0.490
SE	0.918 (0.870, 0.966)	-0.018 (0.025)	-0.140, 0.103	0.038
CI width	0.607 (0.424, 0.791)	-0.17 (0.749)	-1.647, 1.289	0.014
**Second outcome: Continuous (*n* = 10)**				
β	0.994 (0.979, 998)	0.292 (0.511)	-0.710, 1.293	0.074
SE	0.957 (0.886, 0.984)	-0.331 (0.669)	-1.642, 0.980	0.017
CI width	0.957 (0.886, 0.984)	-1.289 (2.626)	-6.436, 3.859	0.017

*CCC* Concordance correlation coefficient, *LOA* Bland and Altman limits of agreement, *B-B* Bradley-Blackwood

**Fig. 1:**
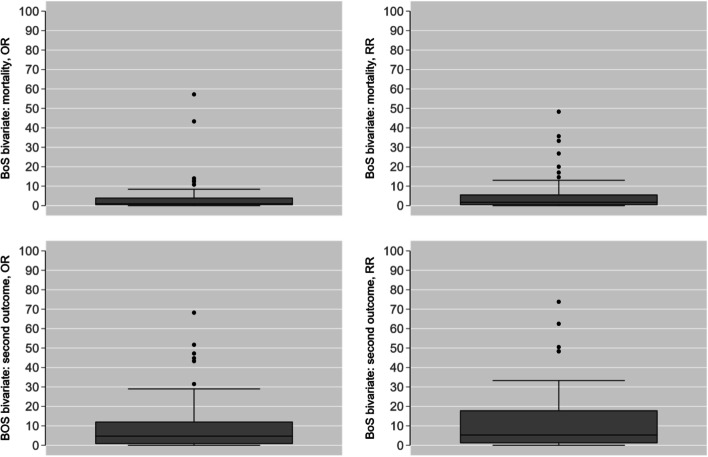
Bivariate distribution of BoS for OR (left) and RR (right)

The bivariate distributions of BoS are displayed in Fig. 1, where an increment of BoS for RR compared with OR, for both mortality and the second outcome is evident.

**Fig. 2:**
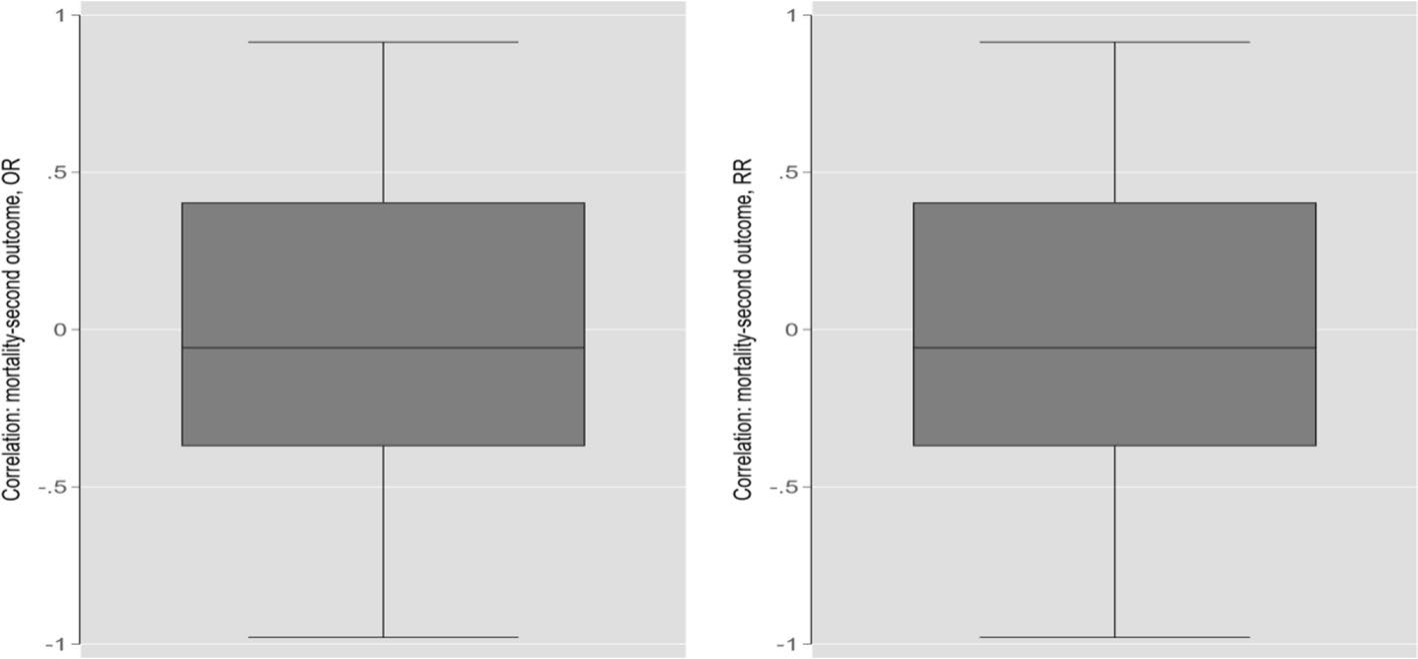
Bivariate correlations (mortality: second outcome) for OR (left) and RR (right)

The bivariate total marginal correlations, mortality vs second outcome, are shown in Fig. 2; both metrics displayed similar distribution.

### Tri-variate model: OR

For the 30 meta-analyses, the median (minimum, p25, p75, maximum) number of studies per meta-analysis for the primary mortality outcome was 13(4, 9, 16, 24); for the second outcome 8(4, 6, 10, 20); and the third 7(3, 5, 10, 15). In only 2 meta-analyses [37, 49] was there equality between the reported primary, second and third outcome study numbers. In the MVMA the “bscovariance” option was used on 13 occasions [37, 38, 46, 54, 57, 62, 66, 72, 73, 77, 78] and there were 5 instances of “large” *Z* values [37, 54, 72, 77, 78] which were sufficient to render estimates implausible and they were not further considered (median number of RCT per meta-analysis for primary, second and third outcomes 12, 6 and 5 respectively). The outcome data set was thus 25 meta-analyses. Second outcomes were binary in 18 and continuous in 7.; third outcomes were binary in 6 and continuous in 19; the “bscovariance” option being used in eight cases.

Concordance estimate analysis is given in Table 6. Variable concordance between uni- and multivariate estimates was observed. Multivariate estimate precision (SE) increased, and confidence interval width tended to decrease compared with univariate, across and within outcomes. A tendency for concordance between means and variances was apparent. Reversal of coefficient estimate significance, univariate versus MVMA, occurred in two cases for mortality ([38, 73] non-significant to significant, one meta-analysis exhibiting small study effects [38]); nine cases for second outcomes (significant to non-significant in 3 [67, 69, 79], one meta-analysis exhibiting small study effects [67]; non-significant to significant in 6 [37, 57, 59, 62, 66, 73]) and 7 cases for third outcomes (significant to non-significant in 3 [40, 46, 67], one meta-analysis exhibiting small study effects [67]; non-significant to significant in 4 [37, 38, 69, 73] with one demonstrating small study effects [37]).

**Table 6:** Concordance analysis for trivariate model (OR): univariate versus multivariate

	**CCC (95%CI)**	**Difference (SD)**	**95%LOA**	**B-B F-test**
**OR**				
**Mortality**				
β	0.775 (0.631, 0.919)	-0.008 (0.119)	-0.241, 0.225	0.051
Mortality SE	0.839 (0.747, 0.931)	0.031 (0.103)	-0.172, 0.233	0.0001
CI width	0.895 (0.844, 0.945)	0.091 (0.542)	-0.972, 1.154	0.0001
**Second outcome: Binary (*n* = 18)**				
β	0.378 (-0.027, 0.783)	0.056 (0.246)	-0.426, 0.537	0.588
SE	0.460 (0.199, 0.720)	0.004 (0.130)	-0.251, 0.259	0.002
CI width	0.452 (0.177, 0.726)	-0.090 (0.662)	-1.387, 1.207	0.003
**Second outcome: Continuous (*n* = 7)**				
β	0.678 (0.128, 0.909)	-1.386 (4.851)	-10.894, 8.122	0.264
SE	0.615 (0.078, 0.875)	1.005 (1.559)	-2.051, 4.060	0.104
CI width	0.634 (0.121, 0.880)	3.844 (5.904)	-7.729, 15.416	0.085
**Third outcome: Binary (*n* = 6)**				
β	0.597 (-0.185, 916)	-0.327 (0.570)	-1.444, 0.790	0.510
SE	0.776 (0.058, 0.965)	0.004 (0.108)	-0.207, 0.215	0.991
CI width	0.782 (0.073, 0.966)	0.021 (0.437)	-0.835, 0.878	0.980
**Third outcome: Continuous (*n* = 19)**				
β	0.708 (0.646, 0.771)	-1.253 (7.797)	-16.35, 14.030	0.0001
SE	0.819 (0.688, 0.950)	0.098 (30,307)	-6.384, 6.579	0.047
CI width	0.813 (0.678, 0.947)	0.510 (13.136)	-25.237, 26.257	0.043

Estimates for the second outcome, continuous and third outcome, binary were tentative due to the low n, but are included for completeness

*CCC* Concordance correlation coefficient, *LOA* Bland and Altman limits of agreement, *B-B* Bradley-Blackwood

### Tri-variate model: RR

Of the 30 tri-variate meta-analyses, there was one instance of complete convergence failure [46] and seven instances of “large” *Z* values [37, 38, 56, 66, 72, 73, 78] which were sufficient to render estimates implausible (median number of RCT per meta-analysis for primary, second and third outcomes 10, 6 and 5 respectively); the outcome data set was thus 22 meta-analyses [37, 40, 47, 49, 50, 53–55, 57, 59, 60, 62, 67–69, 71, 76, 77, 79, 80]. The median (minimum, maximum) number of studies per meta-analysis for the primary mortality outcome was 13(4, 24); for the second outcome, 8(4, 20); and the third 7(4, 15). Second outcomes were binary in 15 and continuous in 7; third outcomes were binary in 7 and continuous in 15. In the MVMA the “bscovariance” option was used on 3 occasions [57, 62, 67]. Concordance estimate analysis is given in Table 7. Concordance between uni- and multivariate estimates was uneven, with no consistent relative change in multivariate estimates, compared with univariate, across or within outcomes. A tendency for concordance between means and variances in second and third outcomes was apparent. Reversal of coefficient estimate significance, univariate versus MVMA, occurred in two case for mortality ([37, 62] with no small study effects, non-significant to significant, not concordant with the OR cases); five cases for second outcomes with no small study effects (significant to non-significant in 2 [69, 79], concordant with OR cases; non-significant to significant in 3 [54, 57, 77]; concordant with one OR cases only [57]); and 3 cases ([54, 60, 67] non-significant to significant, one exhibiting small study effects [67]) for third outcomes with no concordance with OR cases.

**Table 7:** Concordance analysis for trivariate model (RR): univariate versus multivariate

	**CCC (95%CI)**	**Difference (SD)**	**95%LOA**	**B-B F-test**
**RR**				
**Mortality**				
β	0.438 (0.145, 0.722)	-0.028 (0.186)	-0.393, 0.337	0.011
Mortality SE	0.009 (-0.241, 0.257)	-0.241 (2.040)	-4.295, 3.703	0.0001
**Second outcome: Binary (*n* = 15)**				
β	0.352 (0.025, 0.679)	-0.068 (0.321)	-0.697, 0.562	0.007
SE	0.051 (-0.263, 0.365)	-0.045 (0.197)	-0.430, 0.340	0.001
CI width	0.113 (-0.271, 0.498)	-0.345 (1.303)	-2.898, 2.898	0.018
**Second outcome: Continuous (*n* = 7)**				
β	0.702 (0.180, 0.916)	-0.769 (4.669)	-9.920, 8.381	0.282
SE	0.923 (0.629, 0.986)	0.129 (0.977)	-1.785, 2.043	0.903
CI width	0.929 (0.812, 0.987)	0.246 (3.773)	-7.149, 7.641	0.848
**Third outcome: Binary (*n* = 7)**				
β	0.681 (0.094, 0.917)	-0.074 (0.464)	-0.984, 0.836	0.397
SE	0.508 (-0.133, 0.849)	0.063 (0.209)	-0.347, 0.473	0.283
CI width	0.475 (-0.149, 0.829	0.332 (0.926)	-1.484, 2.148	0.229
**Third outcome: Continuous (*n* = 15)**				
β	0.930 (0.813, 0.973)	-0.174 (0.635)	-1.484, 1.077	0.413
SE	0.850 (0.740, 0.960)	0.015 (0.743)	-1.433, 1.473	0.016
CI width	0.849 (0.739, 0.959)	0.123 (2.933)	-5.625, 5.871	0.015

Estimates for the second outcome, continuous and third outcome, binary are tentative due to the low n, but are included for completeness

*CCC* Concordance correlation coefficient, *LOA* Bland and Altman limits of agreement, *B-B* Bradley-Blackwood

**Fig. 3:**
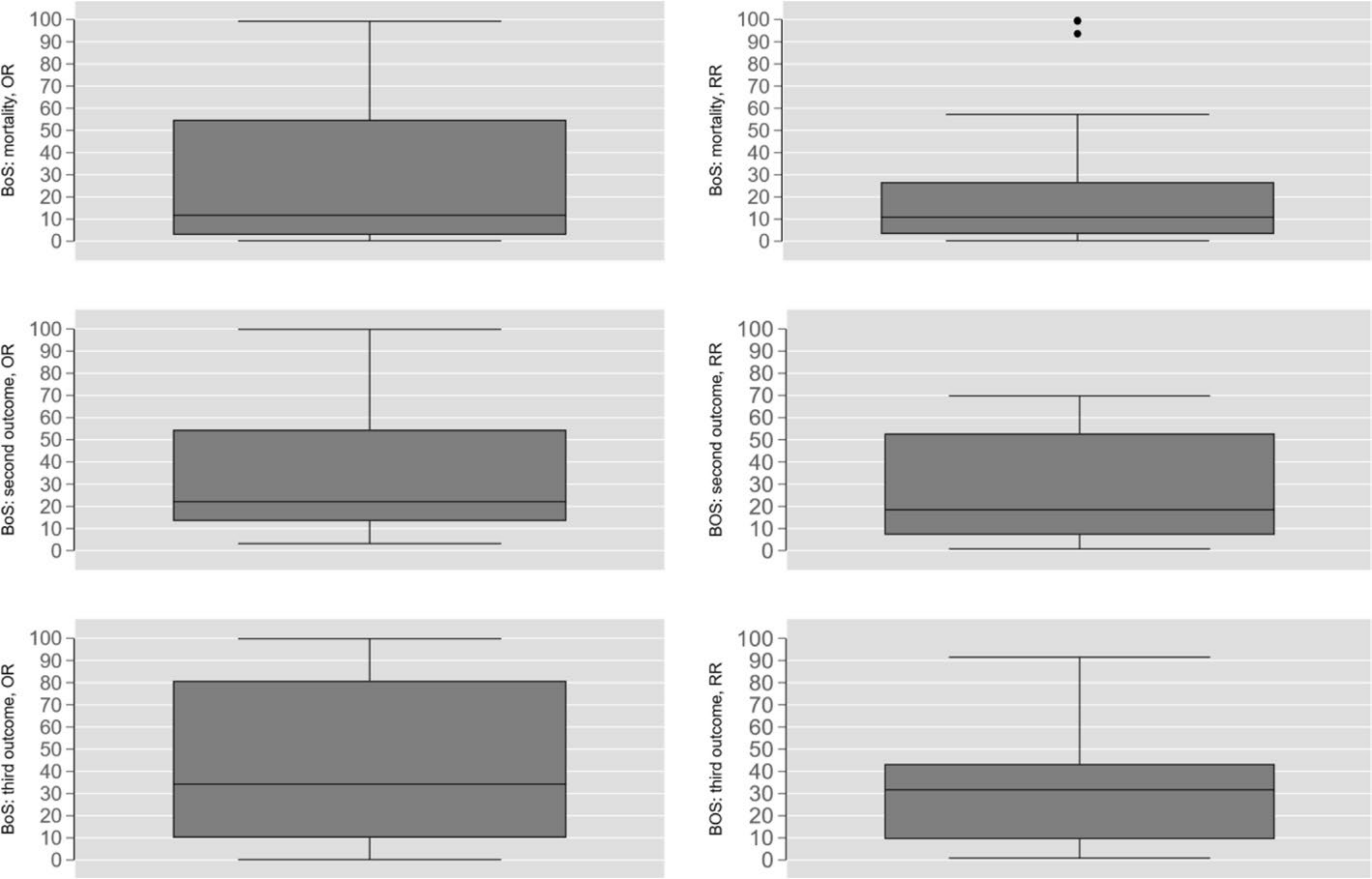
Tri-variate distribution of BoS (mortality, second and third outcomes) for OR (left) and RR (right)

The tri-variate distributions of BoS are displayed using boxplots in Fig. 3. The increment of BoS for OR compared with RR for mortality and the third outcome is evident. In the panel (right top) showing BoS mortality RR there were points of large BoS for two MVMA meta-analyses, 99.3 and 93.6 [57, 62]. Both these MVMA utilized the “bscovariance” option of “mvmeta” as there was initial unresolved convergence. The estimated between study mortality variance was minimal for both (5.24e-06 and 0.005, respectively) and the status of these estimates may be circumspect.

**Fig. 4:**
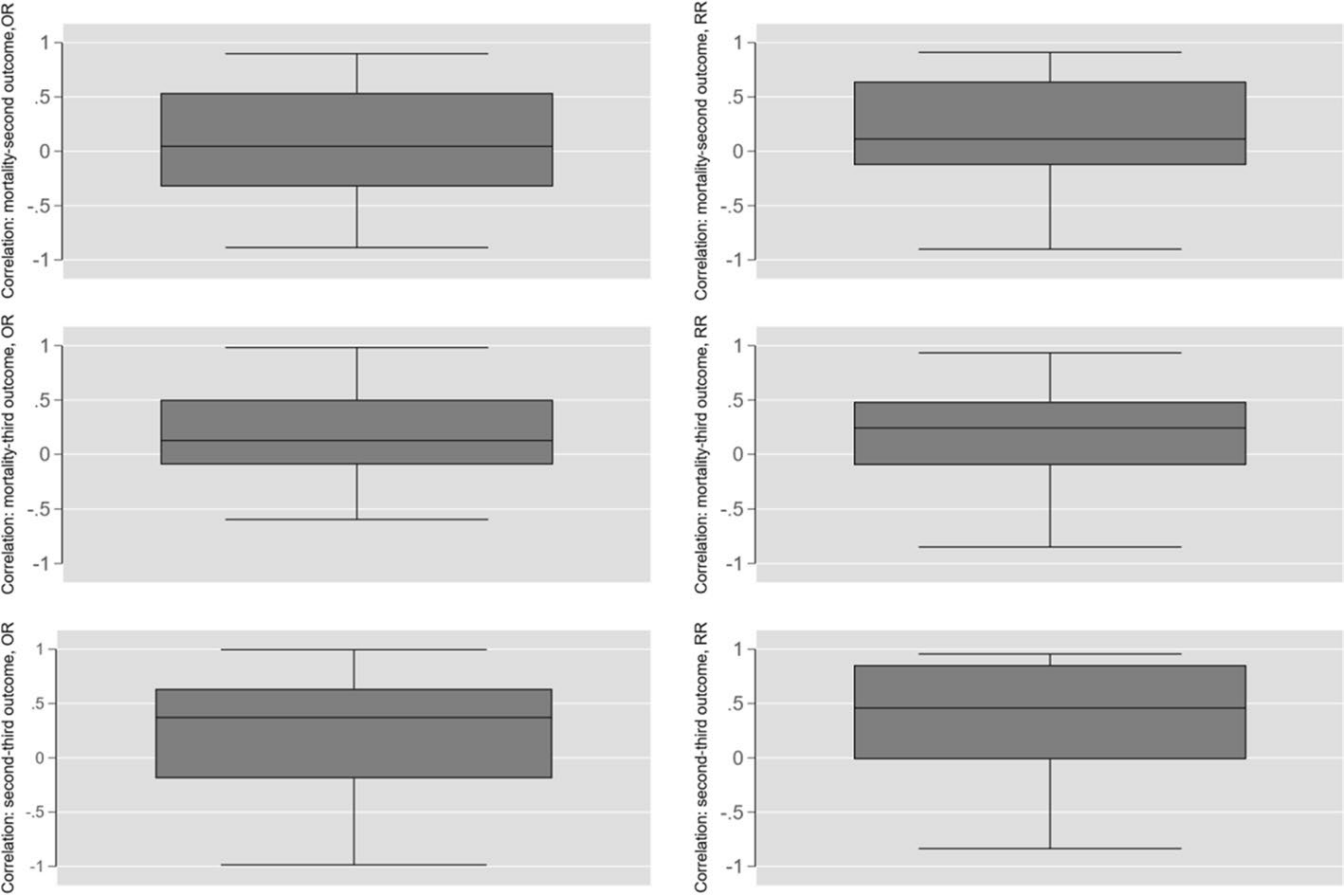
Tri-variate total marginal correlations (mortality-second outcome, mortality-third outcome, second-third outcome) for both OR (left) and RR (right)

The tri-variate total marginal correlations for both OR (left) and RR (right)are shown via boxplots in Fig. 4; with progressive movement to positive correlations from mortality-second outcome through second-third outcome. Positive correlations appeared more frequent with the RR metric.

## Discussion

It is easy to forget that the MVMA approach has a long history dating back to at least 1993 [81] and has subsequently been formally implemented in popular statistical software packages [82–85]. This being said, MVMA still appears rarely used by practitioners, a decade after a 2009 review by Riley [1]. From within the social science paradigm Becker, in 2000, pointed out that ignoring outcome dependence in meta-analysis will affect Type I error rates and precision and bias of estimates: “No reviewer should ever ignore dependence among study outcomes” [82]. In the current study the total marginal correlations for both bi- and tri-variate analyses was sizeable overall and, depending upon the composition of the non-primary outcomes, more positive than negative and more so for the tri-variate case.

One of the principal attractions of MVMA is estimation of the BoS between parameters, well demonstrated in Fig. 3. Most of the BoS would appear to derive from studies which are more “atypical” in design. In particular, the BoS of secondary outcomes of the *i*th study is a function of the within-study variance matrix $\left( {V_{i} } \right)$ and the harmonic average $\bar{V}$ of all the $V_{i} {\text{s}}$. BoS can only arise if there are differences between the $V_{i} {\text{s}}$; which would entail studies of”..substantive difference[s] in background and research methods…”, not simply different sample sizes [10]. The magnitude of outcome BoS would appear to be bounded by percentage of missing data for that outcome [6, 24], which in the current study was substantial (see Results). A percentage missingness of 30–35% of studies informing an outcome was found to result in a “more pronounced” BoS in one empirical study [14]. Any nexus between BoS and missingness requires a missing at random (MAR) assumption, as opposed to missing completely at random (MCAR) for univariate meta-analysis [21]. The notions of MAR and MCAR are well recognised in the bio-medical literature [86], albeit inconsistency of usage has been documented [87]; in particular, the conflation of (non)”ignorable” and MAR [22]. Perhaps not surprisingly, within the domain of outcome reporting bias (ORB) [4], MVMA has been a method of choice to investigate the impact of ORB upon meta-analytic conclusions [22, 88].

Computationally, MVMA requires both within- and between-study correlations and the former are typically not known and are likely not to be available, especially in higher order (trivariate) models [24]. Riley provided four alternate methods to overcome these problems [1]; the most straightforward, yet laborious, being a sensitivity analysis by correlation imputation over the entire range (-1 to + 1). Riley’s alternate model [15] has been found to have good asymptotic statistical properties compared with a fully hierarchical REML model, with known within-study correlations, and with separate univariate meta-analyses. The performance may be problematic when the overall correlation $\left( {\hat{\rho }} \right)$ is very close to 1 or -1. In the current study, only two instances were found; in the bivariate RR MVMA, $\hat{\rho }$ = 0.999 [75], and the trivariate OR MVMA, $\hat{\rho }$ = -0.986 ([57], second versus third outcome); both MVMA utilised the “bscov” option. As the Riley model is a “working” model when the true data generating mechanism is a RE model, the standard variance estimates may not provide confidence interval coverage at the nominal level [21]. Complete failure of convergence in the current study was rare, occurring in one instance [46], but problematic SE estimation was exhibited in the trivariate series, 5 instances in OR metric and 7 in RR. This may relate to the small number of RCT in second and third outcomes (see Table 8), but these numbers were not substantially different compared with meta-analyses not demonstrating this feature, as shown in Table 8.

**Table 8:** Number of studies per meta-analysis (minimum, median and maximum), a propos large z values

	Mortality	Second outcome	Third outcome
OR			
Acceptable z	4, 12 & 24	4, 8 & 20	3, 7 & 15
Large z	6, 13 & 16	5, 6 & 9	4, 5 & 10
RR			
Acceptable z	4, 12.5 & 24	4, 9 & 20	4, 7 & 15
Large z	7, 10 & 16	4, 6 & 9	3, 7 &15

Frequentist and Bayesian empirical comparisons between univariate meta-analyses and MVMA have appeared in the literature [8, 14, 89–93] with results demonstrating similar (pooled) parameter estimates between the two analytic forms. However, papers by Riley and co-workers [1, 15, 24, 94], which included formal simulation studies, found advantage; a smaller standard error and mean-square error of pooled estimates, predicated upon the presence of missing data; again, assuming missing at random. That is, in the presence of complete data a bivariate analysis would not be expected to produce a gain in statistical efficiency. The extension to trivariate and higher order outcome data and the inability to provide within study correlations was thus identified as a “pressing research issue”; to wit, the “alternative” model of Riley [15]. Price et al. suggested that estimates of clinical and /or statistical conclusions from MVMA may occasionally differ from those from univariate analyses and observed, somewhat wryly, that any claimed discrepancy “…says more about the dangers of using concepts of statistical significance than it does the use of MVMA” [8]. The results from the current analysis were somewhat at odds with these sentiments and with the general results of bivariate studies, both empirical and simulation (see below), albeit the caution about the variance estimates of the Riley model, above, are noted. A variable change in the multivariate precision of primary mortality outcome estimates compared with univariate analysis was present for both bivariate and tri-variate meta-analyses and for metric. For second outcomes, precision tended to decrease, and CI width increase for bivariate meta-analyses; for third outcomes, precision increased, and CI width decreased. The latter finding appears not to have been previously reported although analytic reports of the tri-variate structure are rare; one case only reported by Price et al. study [8] and two by Trikalinos et al. [14]. With respect to the observed relative changes (univariate versus multivariate) across four concordance analyses, the magnitude of the difference was rather small and accompanied by a more substantial SD, suggesting a heterogeneity of the MVMA effect, grounded in the individual meta-analyses and dependent upon the nature of the outcome, binary or continuous. As MVMA allows for correlation between outcomes, CIs may be wider on the basis of increased between-study variance [8], but this was observed only in the bivariate case in the current analysis. The experience of Price et al. that “MVMA methods can be applied only in a minority of reviews of interventions in pregnancy and childbirth” [8] was not consistent with the current study.

A reviewer pointed to the wide LOA of the β estimates for the second continuous outcome (days) in Table 6 (trivariate OR MVMA), this being -10.894, 8.122. Of the seven meta-analyses considered, two had stand-out differences between univariate and MVMA estimates; the study of Wan et al. ([57], intra-meta-analytic study number = 4), -11.31, -18.17 and Chen et al. ([69],intra-meta-analytic study number = 10), -11.27 and -4.26. The former study used the “bscov” option, recording a BOS for the second outcome of 54% and correlation between second and third outcomes of 0.986; the latter had normal convergence but record a BOS for the second outcome of 92.4% with a correlation between second and third outcomes of 0.995. This may be indicative of problematic estimation, which has been mentioned above and further addressed in “Limitations”, below. Trikalinos et al. ([14], point 4.1), using Bayesian methods, observed that “Generally, point estimates are comparable”; Price et al. ([8], Table 2) using “mvmeta” recorded differences in β between univariate and MVMA, but did not focus attention on such; and in the bivariate simulation study of Riley et al. ([94], Table 4), bias of the mean for $\beta _{1} \,{\text{and}}\,\beta _{2}$ was comparable with coverage for both between 93–98%; similar results were also observed when considering the “alternate” model of Riley ([15], Table 1).

The differences between the results of the current study and those referenced above [8, 14, 89–93] needs some further explication with regard to data structure. The bi- and tri-variate meta-analyses under consideration were relatively conventional; a primary mortality outcome and second and third recorded outcomes which were not direct extensions of the primary outcome. For second and third outcomes, both categorical (binary) and continuous outcomes were considered, unlike Trikalinos et al. [14] where outcomes were categorical. No repeated measures of a primary outcome, such as different mortality time-points or different types of mortality (all cause or disease specific) were considered; the latter structure featured in the studies of Trikalinos et al. [14, 91], Arends [93] and also in an empirical example Riley et al. [15]. Within the critical care domain the use of MVMA analysis with different mortality time-points has been recently presented [95].The current study did not focus on the impact of different meta-analytic estimators as in Berkey et al. [92], generalized least squares and multivariate maximum likelihood, nor adopt the Bayesian framework of Trikalinos [14]. That bivariate models have been used in systematic reviews of diagnostic test studies for some years, was noted in 2009 by Riley and both Simel et al. [90] and Dahabreh et al. [89] found little advantage for bivariate approaches when considering estimates of sensitivity and specificity. With respect to the change of estimate significance reported here, univariate versus MVMA, the use of the MVMA “bscov” option may have been consequential. For the OR metric, where 24 significance changes occurred, there were seven instances [37, 46, 57, 62, 66, 69, 73], all in trivariate MVMA. For the RR metric, again with the trivariate data structure, there were three [57, 62, 67].

**Fig. 5:**
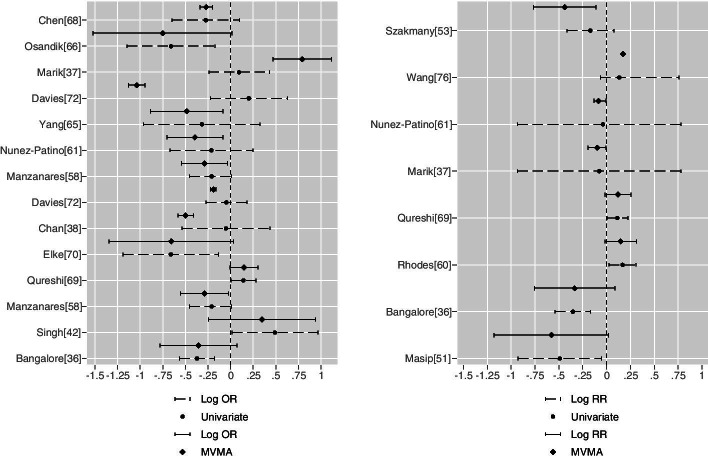
Binary outcome variables (OR left panel, RR right panel): univariate versus MVMA as couplets. For OR: Bangalore [36], Singh [43], Manzanares [59], Qureshi [70] and Elke [71], second outcome bivariate meta-analysis; Chan [38] and Davies [73], mortality tri-variate meta-analysis; Manzanares [59], Nunez-Patino [62]and Davies [73], second outcome trivariate meta-analysis; Marik [37], Osandik [67] and Chen [69], third outcome tri-variate meta-analysis. For RR meta-analysis: Masip [52], mortality bivariate meta-analysis; Bangalore [36], Rhodes [61] and Qureshi [70], bivariate meta-analysis; Marik [37] and Numez-Patino [62], mortality tri-variate meta-analysis; for Wang [77] and Szakmany [54], trivariate meta-analysis, second outcome

**Fig. 6:**
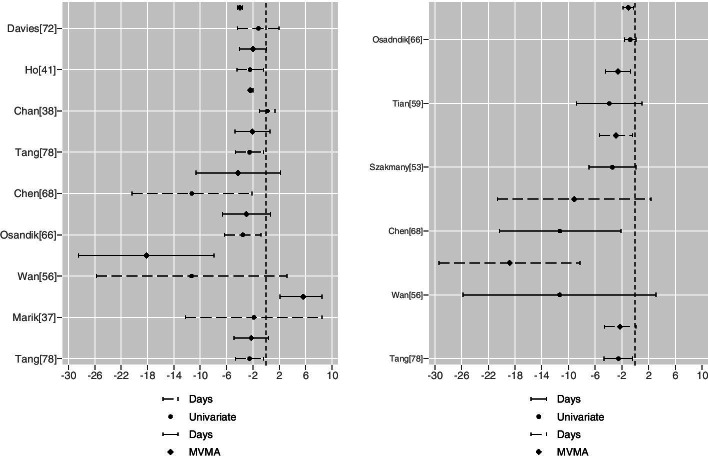
Continuous outcome variables (OR metric left panel, RR metric right panel): the scale is integer days (one case (OR [46]) reporting blood loss in ml was omitted due to scaling incompatibilities). For the (OR) left panel: Tang [79] bivariate meta-analysis; Marik [37], Wan [57], Osandik [67], Chen [69] and Tang [79] second outcome tri-variate meta-analysis; Chan [33], Ho [39] and Davies [73] third outcome tri-variate meta-analysis. For the (RR) right panel: Tang [79], Wan [57] and Chen [69] second outcome tri-variate meta-analysis; Szakmany [54], Tian [60] and Osandik [67] third outcome tri-variate meta-analysis

These changes of statistical significance are shown in forest plots as couplets, univariate versus MVMA, for binary (null line unity, Fig. 5) and continuous (null line zero, Fig. 6) outcomes. A majority of the CI width changes that achieved a change of significance about the null appear substantial; the clinical import of such changes would require case by case determination [96].

Disparities between the OR and RR occurred over a range of indices and may be a function of the current cohort. However, OR and RR are not merely interchangeable metrics and there is no monotone relationship between them [16]. Recent papers have drawn attention to potential estimation problems with the RR. First, the RR effect magnitude is dependent upon the underlying baseline prevalence, shifting toward 1 as prevalence increases, and is a ratio of two conditional probabilities, whereas the OR is a likelihood ratio whose magnitude reflects the fold increase in odds, baseline to intervention, independent of prevalence [97]. Second, under both the DerSimonian-Laird [98] and REML formulations, the requirements of log(RR) estimation to be compatible with study level event rates in the [0,1] interval $(\pi _{j} {\text{treat}}\,{\text{ < }}\,{\text{1}}\,{\text{and 0}}\, < \,\pi _{j} {\text{control}}\,{\text{ < }}\,{\text{1)}}$ demand restriction on the parameter space with ensuant bias in estimates of both $\tau ^{2}$ and log(RR). Thus risk relativism may be an “illusion “ [97] and the OR “appears to be a safer option” [99]. This being said, Xiao and colleagues argued that interpretability issues of the OR, lack of collapsibility and a dependence on the baseline risk, negates any in-principle recommendation for the OR [100].

## Limitations

The current study utilised a single meta-analytic cohort from the critical care domain and had a modest number of bivariate meta-analyses, but less so in the trivariate series. The preference for the alternate model of Riley was a potential limitation, but when reviewing a number of bivariate and tri-variate studies in two metrics the use of sensitivity analysis by specifying within study correlations (via the “wscor” option of “mvmeta”) would be unwieldy and potentially uninterpretable. This being said, the recommendation of Riley et al. in the landmark 2008 paper [15], was that in the presence of overall correlations > 0.9 in absolute value, practitioners “..should assess the robustness of pooled results to small changes in $\hat{\rho }$ as a sensitivity analysis”. In the MVMA where large z values were found and subsequently not considered, for the OR studies [37, 54, 72, 77, 78] and for the RR studies [37, 38, 56, 66, 72, 73, 78], all meta-analyses had $\hat{\rho }$ > 0.9 in at least one of the correlations. Whether such a modus operandi would yield credible z values and pooled estimates has not been explored.

The current study has adopted a workable and practical solution to the particular requirements of MVMA. Future studies should replicate or otherwise the findings in this paper using the “alternate” meta-analytic model of Riley and consider meta-analyses from specific disciplines, moving beyond the bivariate data structure to encompass “…three or more end points…” [1], albeit such estimation may be challenging.

## Conclusions

MVMA elucidates the structure and correlation between multiple reported outcomes in univariate meta-analyses and resolves outcome reporting bias. Change in estimate precision and CI width with MVMA appeared context dependent. The BoS entailed in this technique may be quantified and change of parameter significance may be a consequence. MVMA is a feasible solution to the meta-analytic estimation of multiple univariate effects.

## Data Availability

The collection of the original data set (current reference [9]) from published papers was the joint work of the author (John L Moran) and Dr Petra Graham (see below) and ownership of the data resides with both persons and is thus not available in the public domain, nor in repository.
